# Outcome Prediction by Diffusion Tensor Imaging (DTI) in Patients with Traumatic Injuries of the Median Nerve

**DOI:** 10.3390/neurolint16050078

**Published:** 2024-09-19

**Authors:** Théa Voser, Manuel Martin, Issiaka Muriset, Michaela Winkler, Jean-Baptiste Ledoux, Yasser Alemán-Gómez, Sébastien Durand

**Affiliations:** 1 Department of Plastic and Hand Surgery, Lausanne University Hospital, 1005 Lausanne, Switzerland; thea.voser@hin.ch (T.V.); michaela.winkler@hin.ch (M.W.); 2 Department of Diagnostic and Interventional Radiology, Lausanne University Hospital, 1005 Lausanne, Switzerland; manuel.hr.martin@gmail.com (M.M.); jean-baptiste.ledoux@chuv.ch (J.-B.L.); yasser.aleman-gomez@chuv.ch (Y.A.-G.); 3 Department of Ergotherapy, Lausanne University Hospital, 1005 Lausanne, Switzerland; issiaka.muriset@chuv.ch

**Keywords:** diffusion tensor imaging, MRI, peripheral nerve trauma, peripheral nerve surgery, median nerve

## Abstract

**Background/Objectives:** The accurate quantification of peripheral nerve axonal regeneration after injury is critically important. Current strategies are limited to detecting early reinnervation. DTI is an MRI modality permitting the assessment of fractional anisotropy, which increases with axonal regeneration. The aim of this pilot study is to evaluate DTI as a potential predictive factor of clinical outcome after median nerve section and microsurgical repair. **Methods:** We included 10 patients with a complete section of the median nerve, who underwent microsurgical repair up to 7 days after injury. The follow-up period was 1 year, including the current strategy with clinical visits, the Rosén–Lundborg score and electroneuromyography. Additionally, DTI MRI of the injured wrist was planned 1, 3 and 12 months post-operatively and once for the contralateral wrist. **Results:** The interobserver reliability of DTI measures was almost perfect (ICC 0.802). We report an early statistically significant increase in the fractional anisotropy value after median nerve repair, especially in the region located distal to the suture. Meanwhile, Rosén–Lundborg score gradually increased between the third and sixth month, and continued to increase between the sixth and twelfth month. **Conclusions:** DTI outcomes three months post-operation could offer greater predictability compared to current strategies. This would enable faster decision-making regarding the need for a potential re-operation in cases of inadequate early reinnervation.

## 1. Introduction

Peripheral nerve traumatic injuries, including those of the upper arm, are common and frequently affect young patients. The adequate sensory and motor function of the median nerve is crucial to ensure the ability to use the hand [1].

Current strategies after a nerve is surgically repaired include clinical assessment and neurophysiological studies. These strategies are limited, as they are highly clinician-dependent and poorly detect early reinnervation [2,3]. Furthermore, electrophysiological examination, requiring needles to be inserted into the muscles, is an invasive procedure. In the case of poor nerve regrowth, clinical results will be poor. If surgical revision is delayed, the risk of poor results is also high. It is therefore important to know as soon as possible whether the nerve is growing well or whether surgical revision is necessary. Especially in reconstructions in the area of the brachial plexus or lumbosacral plexus, there is a risk of the rupture of nerve sutures in the event of inadequate post-operative immobilization. A novel approach to detect and quantify peripheral nerve regeneration is therefore needed to provide a practical tool for the management of peripheral nerve trauma.

MRI can be used for the qualitative assessment of peripheral nerves [4]. Applying appropriate magnetic field gradients, MRI can be sensitized to the diffusion of water molecules in the direction of the field gradient. This technique is called diffusion-weighted imaging (DWI) [5]. Free water demonstrates Brownian motion of water molecules, and many materials have intrinsic structural properties that hinder diffusion so that diffusivity is greater in some directions than in others. This property is known as diffusion anisotropy. In well-aligned nerve tracts, water diffuses relatively freely along the axis of the tract, but its diffusion is strongly restricted in any other direction. The direction of maximum diffusivity has been shown to coincide with the main fiber tract orientation [6]. The anisotropic diffusion of water molecules in white matter fiber tracts can be limited by several factors such as myelin sheath, axonal membranes, fiber density and packing or pathological changes [7]. When diffusion-sensitizing gradients are applied from multiple directions (at least six), the water diffusion in a voxel can be modeled by a diffusion tensor [6]. This tensor is a 3 × 3 matrix and comprehensively describes diffusion within three-dimensional space, under the assumption of a Gaussian displacement distribution. Typically depicted as either an ellipsoid or an orientation distribution function, it encapsulates the diffusion process, and it is the basis of diffusion tensor imaging (DTI). The ellipsoid comprises three principal eigenvectors $\vec{e}$_1_, $\vec{e}$_2_ and $\vec{e}$_3_ with eigenvalues (λ1, λ2, λ3).

On one hand, these eigenvalues are used to compute different voxelwise metrics such as axial diffusivity (λ1), radial diffusivity ((λ2, λ3)/2), mean diffusivity <λ> = (λ1 + λ2 + λ3/3) and fractional anisotropy (FA):

FA = (3/2)^1/2^[((λ1 − <λ>)^2^ + (λ2 − <λ>)^2^ + (λ3 − <λ>))^1/2^/(λ1^2^ + λ2^2^ + λ3^2^)^1/2^].

These fractional anisotropy maps, derived from DTI acquisition, are expected to be employed as a new diagnostic tool for peripheral nerve injury, as it can evaluate, noninvasively, nerve regeneration not only in a qualitative way but also in a quantitative way [8].

The literature determined that FA values decrease with axonal degeneration and increase with axonal regeneration [8,9,10]. FA changes are correlated with axon number and motor function recovery [8]. An assessment of axonal regeneration in different animal models (rabbits, rats, mice) using DTI was performed, and the correlation between DTI parameters, histology and behavior suggest that DTI can be a potent tool for predicting the extent of functional recovery after peripheral nerve injury in humans.

On the other hand, the eigenvectors computed from diffusion tensors can be used by different tractography algorithms to digitally trace the specific neural pathway of nerve fibers [11].

There have been few isolated reports in humans on the use of DTI and tractography to visualize nerve regeneration following peripheral nerve trauma [12,13,14]. Peripheral nerve DTI has been found to be reliable and reproducible in healthy subjects, with few effects related to the post-processing package used [13].

The aim of this study is to evaluate peripheral nerve DTI as a potential predictive factor of clinical outcome after median nerve microsurgical repair. The validation of peripheral nerve diffusion-derived metrics as an assessment biomarker in peripheral nerve regeneration could be of great importance to the future treatment of a surgical nerve repair. We hypothesize that the DTI outcomes three months post-operation would offer greater predictability compared to current strategies. This would enable faster decision-making regarding the need for a potential re-operation in cases of inadequate early reinnervation.

## 2. Materials and Methods

### 2.1. Subjects and Study Design

This prospective, single-center study, employing analytical and cross-sectional methods, adhered to ethical standards set forth by both institutional and national research committees, in accordance with the principles outlined in the 1964 Helsinki Declaration. Approval for this study was obtained from the Cantonal Research Ethics Committee, Vaud (BASEC-ID 2016-01947), and all patients provided signed written consent prior to participation.

**Figure 1:**
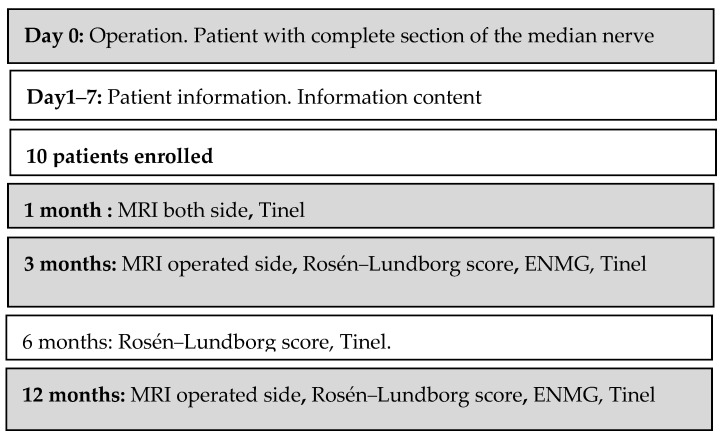
Study design and workflow.

This study included patients with a complete traumatic section of the median nerve at the level of the forearm treated surgically less than 7 days after trauma. Patients with partial median nerve injury or median nerve injury in the hand or elbow were excluded. MRI contraindications were determined by asking patients if they had cardiac implantable electronic devices, implantable neurostimulation systems, cochlear implants, cerebral artery aneurysm clips, magnetic dental implants, hearing aids, piercings, metallic intraocular foreign bodies or any facial injuries with metal, but no patients were excluded because of them. Between 2017 and 2020, this study included 10 patients with a mean age of 38.9 years old (SD 19 years old, 9 males and 1 female). The dominant side was affected in 7/10 cases. The mechanism of trauma was accidental in 7 patients and self-inflicted in 3 patients. A complete section of the median nerve was associated with flexor tendon lesion in all of our cases, radial and/or ulnar artery laceration in 8 cases, ulnar nerve section in 4 cases and radio-carpal ligaments in 1 case. The study design is reported in Figure 1.

### 2.2. Operative Procedure

**Figure 2:**
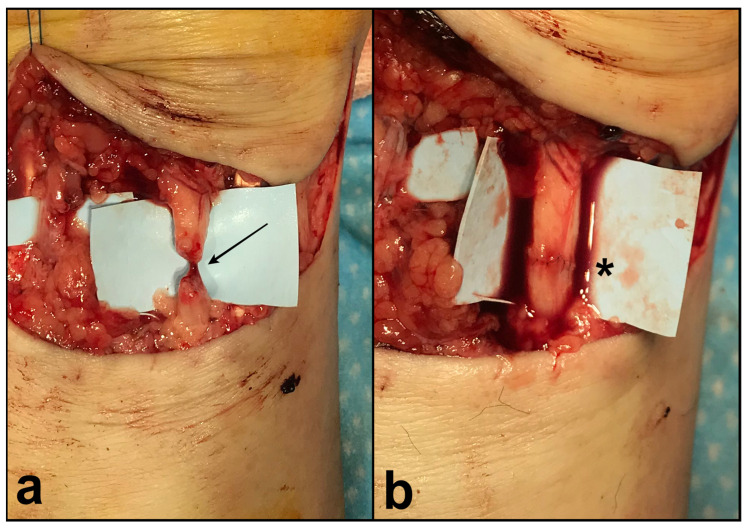
(**a**) Intra-operative photographs (42-year-old female) showing a complete section of the median nerve of the right wrist (black arrow). (**b**) The results after microsurgical suture (black asterisk) with Nylon 10–0 under a microscope and before the adjunction of fibrin glue.

The mean delay for surgery after injury was 0.7 days (SD 0.7 days). The procedure was performed under the axillary nerve block and a tourniquet. Patients with complete median nerve section were identified for recruitment on day 0. The surgery consisted of an end-to-end microscope-assisted surgical suture of the median nerve using a Nylon 9–0 or 10–0 (Ethicon, Inc., Somerville, NJ, USA) with the adjunction of fibrin glue Tisseel VH Fibrin Sealant (Baxter-Immuno AG, Vienna, Austria) (Figure 2). Associated lesions were not considered in the exclusion criteria and were repaired in the same operative time. A sterile dressing and a cast with the wrist in 30° of flexion were applied for 1 month. Rehabilitation sessions with hand therapists were performed, with early active mobilization protocol for associated flexor tendon lesions, paying attention to not compromise the nerve/arterial suture. 

### 2.3. Clinical Assessment

The Rosén and Lundborg Score [15] is a scale system used to document the clinical routine and the quantification of the functional results of patients after median or ulnar nerve repairs on the wrist or distal level of the forearm and includes modules relating to the sensory, motor and pain/discomfort domains. The sensorial domain was evaluated through Semmes–Weinstein monofilaments [16] to test tactile sensitivity. Tactile gnosis was evaluated by two-point discrimination [17], the Shape/Texture Identification (STI) test (Ossur Nordic AB, Uppsala, Sweden) [18] and dexterity according to tasks 4, 8 and 10 of the Sollerman hand function test (mini Sollerman hand function test (Catell AB, Hägersten, Sweden)) [19]. The motor domain was measured by manual muscle strength, graded 0–5 [20], and the strength of grip was measured by a dynamometer (Jamar^®^) [21]. For pain/discomfort, the patient estimated perceived problems about pain/discomfort for cold tolerance and hyperesthesia. The maximum score for each domain was 1.0, with the total score calculated as the sum of the three domains, with a maximum value of 3.0. Thus, the higher the result, the better the hand function is. The measured total score is marked in a graphic with estimated values for “total score” in the shaded area (95% individual prediction interval), thus making it possible to verify if the patient’s recovery conforms to what was expected for the assessment period [22]. The Rosén and Lundborg score and Tinel sign progression according to the scar location was measured at 3, 6 and 12 months by a single hand therapist (I.M.). The Rosén and Lundborg score was chosen because of its high reliability and validity in assessing nerve function. The scale takes into account multiple tests, and the effect of confounding variables in any one test is minimized. However, to calculate the entire score, multiple tests have to be run, which can place a burden on both clinicians and respondents.

### 2.4. Image Acquisition and Processing

For each patient, three separate scan sessions were scheduled at three distinct timepoints: 1, 3 and 12 months following the surgical procedure for the injured wrist. Additionally, a single scan session was conducted during follow-up for the contralateral healthy wrist. Three regions of interest were explored: the region of the suture of the median nerve, the proximal region (3 cm proximal to the suture) and the distal region (3 cm distal to the suture).

Each scan session was performed using a 3-Tesla MRI scanner (Magnetom PRISMA, Siemens Healthineers, Erlangen, Germany) with a maximum gradient amplitude of 80 mT/m, slew rate of 200 T/m/s and a dedicated 16-channel wrist coil. During the whole acquisition, the coil was positioned at the center of the magnet bore. The patient was in the prone position with their hand over their head within the coil and immobilized with cushions, sandbags and bandages.

Three different images were acquired in each scan session (see image acquisition parameters in Table 1): a T2-weighted Turbo Spin Echo (TSE) axial sequence with fat suppression used for anatomical reference, diffusion tensor imaging (DTI) using an Echo-planar imaging (EPI) axial sequence with a multidirectional diffusion Weighting (MDDW) mode to obtain automatically different reconstructions such as a combined directionally diffusion image (TRACE), Apparent Diffusion Coefficient (ADC), fractional anisotropy (FA) and Colored Fractional Anisotropy (FA Color), and a three-dimensional T1-weighted Sampling Perfection with Application optimized Contrast using different flip angle Evolution (SPACE) acquired in a coronal plane. DTI parameters were optimized to achieve accurate diffusion imaging with minimal artifacts. A repetition time of 3900 ms and Echo Time of 82 ms allowed for sufficient time for measuring water diffusion in different directions. Voxel size (1 × 1 × 3 mm) provided a good resolution in the axial plane while keeping the slice thickness larger to reduce acquisition time. The number of directions (20) offered a balance between precision and scan time. Fat saturation helped minimize artifacts from fat signals. A high bandwidth (1190 Hz/Px) was used to reduce distortion artifacts, which are common in diffusion sequences like EPI. A short Echo Spacing (0.94 ms) reduced distortion from artifacts associated with the EPI technique.

**Figure 3:**
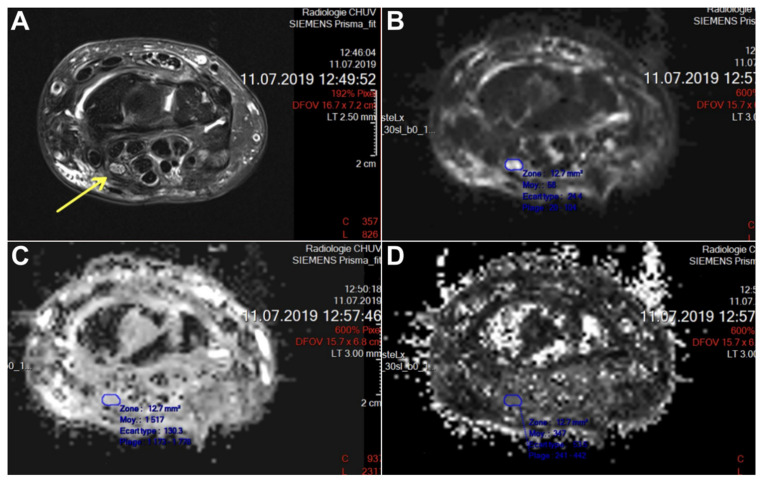
(**A**) The identification of the median nerve (arrow) on the T1-weighted sequence; (**B**) encircling the median nerve on the DWI trace sequence; (**C**) the encircled region of interest copied on the ADC sequence (**D**) and on the FA sequence.

Two independent assessments of the MRI images were performed: First, the identification of the level of the suture of the median nerve was conducted on the T1w image of the injured side. Then, the area of the median nerve on 3 slices centered over the lesion, 3 slices proximal to the lesion and 3 slices distal to the lesion was manually traced (see Figure 3). Each region of interest was mapped to the FA and ADC (Apparent Diffusion Coefficient) images where both the mean FA and ADC values, respectively, were documented for each region of interest. The number of slices between the lesion and the pisiform was counted on the injured side images and reported on the healthy side for each patient.

### 2.5. Electrophysiological Studies

Electroneuromyography was performed at 3 and 12 months post-operatively. The motor response was measured at the thenar and wrist region with electrodes after stimulation proximal to the sutured nerve. The sensory action potentials are sought at digits one to three. The sympathetic skin response was measured with a special device measuring the perspiration of digits one, two and three compared to the contralateral side.

### 2.6. Statistical Analysis

Firstly, the Kruskal–Wallis test was employed to examine whether there were significant variations in Rosén and Lundborg scores across the different timepoints.

Secondly, an Intraclass Correlation Coefficient (ICC) was employed to evaluate the overall agreement between the two raters. This provided a comprehensive understanding of the reliability of measurements obtained by each rater across all regions. Subsequently, to delve deeper into the agreement within individual regions, the Spearman correlation coefficient was computed to evaluate the relationship between the values obtained by both raters for each specific region. Furthermore, a Bland–Altman plot analysis was conducted to visualize any systematic differences or biases between the measurements obtained by the two raters across the regions of interest. These analyses show that the fractional anisotropy measurements from different raters are consistent and reliable, strengthening this study’s results.

Thirdly, a Kruskal–Wallis test was employed to ascertain whether there existed a significant effect of time on the FA values within each rater and region. This non-parametric test was chosen due to its suitability for comparing multiple groups when assumptions of normality and homogeneity of variance are not met. Following the detection of any significant differences across time, pairwise comparisons were conducted using the Wilcoxon signed-rank test. This approach allowed for a detailed examination of the specific pairwise differences in FA values within each rater and region, providing valuable insights into the temporal dynamics of FA measures.

Finally, a similar approach to the preceding one was undertaken to estimate differences in fractional anisotropy (FA) values concerning the healthy contralateral side. This comparison aimed to discern any deviations in FA measures between the regions of interest and their corresponding healthy contralateral counterparts. Initially, within each rater and region, a Wilcoxon signed-rank test was used to evaluate whether there were significant differences in FA values between the regions of interest and their healthy contralateral counterparts. Statistical significance was set at *p* < 0.05.

## 3. Results

### 3.1. Clinical Results

The mean (SD) Rosén and Lundborg scores at 3, 6 and 12 months after surgery were 1.20 (0.42), 1.45 (0.45) and 1.69 (0.46), respectively (*p* = 0.0748).

Post-operative pain was controlled with an oral multimodal analgesic. Neuropathic pain required the introduction of gabapentin (Neurontin) or pregabalin (Lyrica) in 8/10 patients. No complications such as infection, suture leak, bleeding, thrombosis or algodystrophy were observed during follow-up. Psychological instability was observed in three patients.

### 3.2. Diffusion Tensor Imaging (DTI)

(a) Injured side

**Figure 4:**
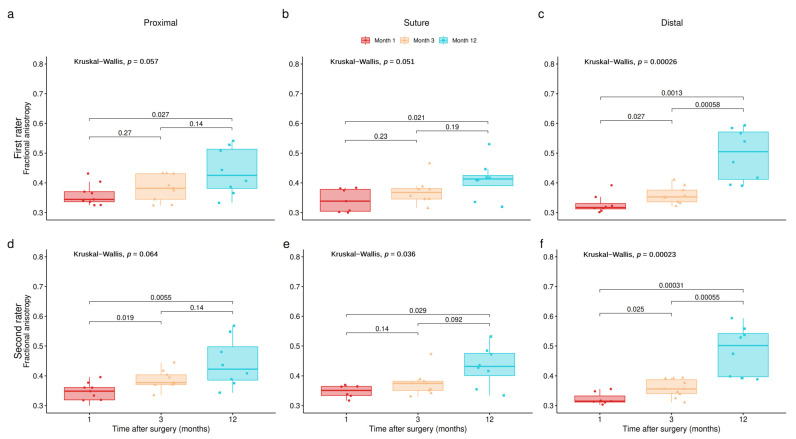
FA values for each timepoint and region of interest. Panels (**a**–**c**) show these differences for proximal, suture and distal regions, respectively, for rater 1. Panels (**d**–**f**) show same results for rater 2.

The M1 and the M3 DTI MRI were performed in all patients (10/10), while the M12 DTI MRI was performed in 8/10 patients. Proximal to the suture, the mean FA values were 0.35397, 0.37237 and 0.43925 at M1, M3 and M12, respectively. At the level of the suture, the mean FA values were 0.32383, 0.35623 and 0.41071 at M1, M3 and M12, respectively. Distal to the suture, the mean FA values were 0.31687, 0.352 and 0.49467 at M1, M3 and M12, respectively (Figure 4).

The mean FA differences were statistically significant between M1 and M12 for all the regions and both raters (see Figure 4, all panels). Differences between M1 and M3 and between M3 and M12 were found by both raters only for the distal region (see Figure 4 panels c and f). The FA increase between M1 and M3 for the proximal and suture regions was present but not statistically significant (see Figure 4a,b,d,e).

(b) Healthy contralateral side

The mean FA values were 0.50313, 0.461 and 0.49038 at the proximal, suture and distal region of the contralateral healthy side according to the lesion of the median nerve on the operated side.

**Figure 5:**
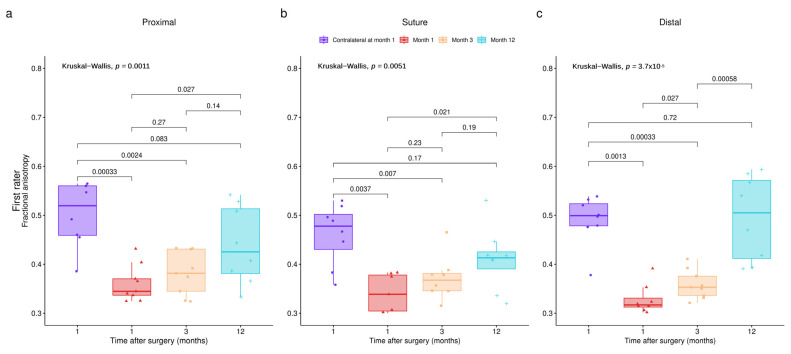
FA between healthy and injured sides for each timepoint at the (**a**) proximal region, (**b**) suture region and (**c**) distal region.

These values of FA were higher than the values of FA on the injured side at M1 and M3 for each level of measurement; these differences were statistically significant (see Figure 4, all panels). The difference between FA on the healthy sides and on the injured sides 12 months post-operatively, for each level of measurement, was not statistically significant (*p* values for the proximal region: 0.16193; suture level: 0.096435; distal region: 0.93731) (Figure 5). At M12, on the injured side, at the proximal, suture and distal levels, the mean FA value reached, respectively, 87.3%, 89.1% and 100.9% of the mean “healthy” FA value (Table 2).

**Figure 6:**
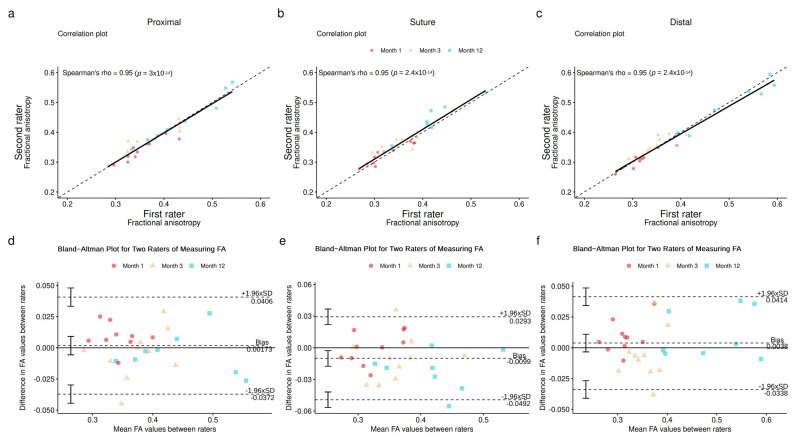
Interobserver agreement according to fractional anisotropy for each region. (**a**–**c**) Correlation plots. (**d**–**f**) Bland–Altman plots.

The interobserver reliability of FA measures was almost perfect (ICC 0.802) (see Figure 6). It was not possible to highlight a correlation between FA values and the Rosén–Lundborg score, principally because of the different timepoints used in the protocol (M1, M3 and M12 for MRI and M3, M6 and M12 for the Rosén–Lundborg score). Even with the extrapolation of the M1 timepoint for the Rosén–Lundborg score, the results were not conclusive.

### 3.3. Electrophysiological Results

The M3 ENMG was performed in 6/10 patients, while the M12 ENMG was performed in 9/10 patients. The motor response in the thenar muscle after proximal stimulation was absent at M3 in 5/6 patients. In 1/6 patients, an electrical and clinical response (normal motor latency but lower amplitude, M4-graded strength) was obtained in the abductor pollicis brevis. The presence of a nerve anatomical variation in the forearm or the hand was probably the origin of this signal. The motor electrical signal at M12 was present in 7/9 patients. The two patients without an electrical response were the patients who performed the second ENMG too early, at 6 and 7 months post-operatively. Sensory action potentials sought in digits I-III were found 3 months post-operatively in 4/6 patients and at the second ENMG in 7/9 patients.

## 4. Discussion

Over the last few years, DTI for peripheral nerve evaluation has undergone significant advancements. These advancements have offered novel insights into clinical practice. By establishing normative values of DTI MRI for specific nerves and locations, clinicians can better discern pathological deviations. This enhances diagnostic accuracy and improves prognostic assessment in peripheral nerve disorders. In a recent meta-analysis [23] which focused on the normal diffusion tensor imaging (DTI) values of the upper limb (median, ulnar and radial nerve), comprehensive insights into baseline DTI metrics were provided. This study offers a reference for interpreting DTI findings in clinical practice. Other studies [24,25] evaluated the DTI of the brachial plexus, providing estimated FA normative values and producing a cartography of the plexus brachial. However, all the authors highlight the fact that diffusion MRI metrics are contingent upon experimental conditions and underscore the necessity of robust harmonization techniques to mitigate non-biological variability.

The MRI with DTI is not currently a part of the management after the surgical repair of the median nerve and was proposed as a supplement to the current strategy, including clinical follow-up, hand rehabilitation and ENMG. This current strategy does not allow us to follow the progression of nerve regrowth because of several limitations; clinically, Tinel sign progression can be useful but is often inaccurate. Motor function takes months to be observed when a median nerve is sectioned proximal to the wrist crease. A clinical score, for example, the Rosén and Lundborg score, often cannot be conducted before 3 months post-operation, because of discomfort and the risk of different suture leaks. Furthermore, these scores are partly subjective and dependent on the patient’s participation. They are also inconsistent since the intensity of cold intolerance will be different depending on the season during which the accident occurs. In our study, the ENMG did not show a motor response 3 months post-operatively when stimulating the median nerve. We know that this exam is poorly contributive in early stages because of the time needed for myelin to regenerate. DTI is more comfortable and less invasive than other measurement methods, particularly electroneuromyography. We showed that the measurement of FA is reliable and reproducible, with an almost perfect interobserver reliability.

In our study, an early significant increase in the FA values could be observed after a microsurgical nerve suture, especially in the region located distal to the suture. In a small case–control study [26], FA values were significantly reduced in traumatic peripheral nerve injury relative to healthy control nerves, and DTI measures improved after the operation procedure, as we observed in our study. With the first DTI MRI 1 month post-operatively and the second one 3 months post-operatively, it is already possible to judge if a reinnervation is occurring. Based on this observation, DTI MRI may have the potential to serve as a predictive tool for early nerve regeneration, but more evidence is needed to confirm its effectiveness. This could be particularly useful to identify the need for an early re-operation when a stagnation of the FA value at 3 months post-operation is measured, if clinical examination is also in favor (for example, no progression of the Tinel sign). Thus, the final functional prognosis could be better by limiting irreversible muscular atrophy occurring with a late re-operation. In this study, we could not highlight a correlation between the FA values and the Rosén and Lundborg score. However, this score could not be calculated earlier in the protocol because of associated lesions contraindicating some parts of the score (for example, strength testing in the situation of associated tenorrhaphy). We also wanted the MRI to be conducted early in the protocol to see if it could become a predictive tool of early recovery. Further investigation with a larger sample would likely show more significant results of the Rosén and Lundborg score at 3, 6 and 12 months and might demonstrate a correlation between this score and the evolution of DTI parameters. Such a correlation would thus allow for a formal indication for surgical revision to be established. There are several other limitations in our study. A complete section of the median nerve was associated with flexor tendon lesion in all of our cases, radial and/or ulnar artery laceration in eight cases, ulnar nerve section in four cases and radio-carpal ligaments in one case. This heterogeneity of lesions makes the comparison of the final functional clinical result more random with more parameters that can explain poor recovery in some patients. The mechanism of trauma was accidental in seven patients and self-inflicted in three patients. This implies a particular population profile with a lot of psychological and psychiatric comorbidities, complicating the treatment and follow-up. Thirdly, our study is based on a small cohort size. The several requirements that clinical studies must meet are transformed into huge challenges when dealing with rare diseases or trauma like the complete section of the median nerve. Patient recruitment can be difficult. Multicentric studies may be interesting but often mean that there is a slower study start-up because of multiple submissions to ethic committees, multiple study protocol amendments, language barriers and different surgical options depending on surgeons.

The recent literature highlights the burgeoning importance of DTI across various facets of peripheral nerve disorder management. DTI has emerged as a promising tool for identifying and characterizing nerve compression. Two meta-analyses [27,28] examined the standard DTI values of the median nerve and their alterations in carpal tunnel syndrome (CTS). Patients with CTS consistently displayed notably lower FA and higher mean diffusivity compared to controls. The most significant differences between asymptomatic adults and CTS patients were observed at the pisiform and at the hamate level. FA and ADC cut-off values for diagnosing CTS within these precise ROIs were proposed. A recent study [29] comparing DTI values between healthy volunteers and patients with lumbar disc herniation with lumbosacral nerve root compression also showed that the FA value of diseased nerve roots was significantly lower. FA could also be considered a novel objective quantitative indicator of treatment effects and a potential indicator of percutaneous stereotactic radiofrequency rhizotomy effectiveness in trigeminal neuralgia patients [30].

In recent years, DTI has emerged as a valuable adjunct to conventional MRI for delineating the spatial relationships between tumors and peripheral nerves [31], guiding surgical planning [32,33] and optimizing therapeutic outcomes. DTI MRI has been used to help differentiate between an infiltrative process (for ex. neurofibromas) and a displacing process (schwannoma) [34]. The potential use of DT as an imaging biomarker is also suggested in various autoimmune or degenerative neurological pathologies [35,36].

While some authors [28] have found minimal impacts of experimental conditions on FA measurements, most advocate for robust standardization techniques to mitigate non-biological variability in DTI datasets. Although routinely employed in the central nervous system, its application poses greater technical challenges in peripheral nerves (e.g., small diameter, DTI’s spatial resolution, similar contrast to adjacent veins and muscles). Nonetheless, future studies should aim to delineate optimal protocols to solidify normative DTI values. A recent study [37] investigated the establishment of a simplified acquisition model using only three vectors, compared to the typical twenty, focusing on the tibial nerve. According to the authors, this simplified model holds promise for peripheral nerves, which exhibit a distinct unidirectional structure, enabling the acceleration of image acquisition while preserving diagnostic performance. This advancement may promote the practical utilization of DTI in clinical practice.

## Figures and Tables

**Table 1:** Image acquisition parameters.

	T2 Tse FS	DTI	T1 Space
Acquisition Time	4 min 57	7 min 40	3 min 10
Slices	30	30	144
Distance Factor (mm)	0.25	0	0
FOV Read (mm)	87	120	160
FOV Phase (mm)	79	120	136
Voxel Size (mm)	0.3 × 0.3 × 2.5	1 × 1 × 3	0.5 × 0.5 × 0.5
Slice Thickness (mm)	2.5	3	0.5
Repetition Time (ms)	5470	3900	500
Echo Time (ms)	83	82	11
Nex	2	1	1
Flip Angle (deg)	147	x	T1 Var
Fat Suppression	Fat Saturation	Fat Saturation	None
Matrix Size	256 × 230	120 × 120	320 × 320
Pat Mode	None	Grappa2	Grappa3
Flow Compensation	Read	None	None
Echo Spacing (ms)	11.9	0.94	5.68 ms
Bandwidth (Hz/Px)	230	1190	363
Turbo Factor	13	120 (EPI)	42
Number of Directions	x	20	x

**Table 2:** Mean FA (standard deviation) values.

Region	M1	Injured Side M3	M12	Healthy Side
Proximal	0.35397 (0.040)	0.37237 (0.052)	0.43925 (0.079)	0.50313 (0.066)
Suture	0.32383 (0.042)	0.35623 (0.051)	0.41071 (0.065)	0.461 (0.062)
Distal	0.31687 (0.036)	0.352 (0.034765)	0.49467 (0.087)	0.49038 (0.051)

## Data Availability

All data generated or analyzed during this study are included in this article. Further inquiries can be directed to the corresponding author.
